# Neighbourhood natural space and the narrowing of socioeconomic inequality in years of life lost: a cross-sectional ecological analysis of the Scottish Burden of Disease

**DOI:** 10.1136/jech-2022-219111

**Published:** 2022-10-17

**Authors:** Natalie Nicholls, Fiona Caryl, Jonathan R Olsen, Richard Mitchell

**Affiliations:** MRC/CSO Social and Public Health Sciences Unit, University of Glasgow, Glasgow, UK

**Keywords:** spatial analysis, health inequalities, geography

## Abstract

**Background:**

Natural space is associated with reduced risk of, and narrower socioeconomic inequalities in, diseases that affect older populations, and some contributors to premature mortality in younger individuals. Burden of disease measures such as years of life lost (YLL) are influenced by premature poor health and death. We hypothesised some association between natural space and both rates of and inequalities in YLL might be present.

**Methods:**

The outcome data were the YLL component from Scottish Burden of Disease 2016, provided at small-area level (datazone) for males and females under 65 years of age in Scotland, UK. Exposure variables were the percentages of land cover within each datazone defined as ‘natural space’ (NS), and ‘natural space and private gardens’ (NSG). Together with a measure of area income deprivation, these were fitted in a multilevel Poisson model accounting for intra-datazone level variation, and spatial autocorrelation between datazones.

**Results:**

An increased percentage cover of NSG was associated with lower YLL in males (incident rate ratio (IRR) 0.993, 95% credible interval (CrI) 0.989 to 0.997) and females (IRR 0.993, CrI 0.987 to 0.998); each 10% increase of natural space cover was associated with a 7% decrease in the incidence rate. An increased amount of natural space within local areas was associated with reduced disparity in YLL between the most and least income deprived areas.

**Conclusions:**

The health benefits of natural space also apply when indicators sensitive to health events at younger ages are used. An increased amount of natural space within local areas has the potential to reduce the disparity in YLL between the most and least income deprived areas—the ‘equigenic’ effect.

**WHAT IS ALREADY KNOWN ON THIS TOPIC**

- Greater access and exposure to natural space has been shown to have a direct impact on health.
- Measures such as years of life lost (YLL) are particularly effective at detecting the impacts of premature poor health and death.
- The association between natural space and YLL in Scotland, after accounting for deprivation, has not been explored.

**WHAT THIS STUDY ADDS**

- These results indicate that, using a measure (YLL) that should not be impacted much by the presence of natural space, some association is still there—further reinforcing how important natural space is to public health.
- An increased amount of natural space within local areas was associated with lower disparity in YLL between the most and least income deprived areas.

**HOW THIS STUDY MIGHT AFFECT RESEARCH, PRACTICE OR POLICY**

- This study reinforces the evidence that local natural space, and in particular private gardens, has a positive impact on health, and the potential for reducing health inequalities; thus natural spaces and private gardens should be an important feature in any building/development planning.

## Introduction

Many studies have assessed the apparent health benefits to individuals and populations of exposure to natural spaces. There is strong evidence that spending more time in ‘green’ areas is associated with many positive health outcomes, including lower heart rate, improved birth weights and lower mortality rates.^1–4^ However, associations are not consistently positive, which may be due to inconsistency in how ‘greenness’ is measured.^1 3 5^ Further, emerging evidence suggests that ‘blue’ spaces provide health benefits, such as lower prescribing of antidepressant medication among older people.^6^ A potential way to advance is therefore to capture *the whole natural environment*, or ‘natural space’, by including both green and blue spaces measured with landcover data.

When epidemiologists assess associations between exposures and health, they often use direct measures of illness or function such as mortality rates or prevalence of poor mental health, and the overwhelming majority of epidemiological studies of natural space take this approach (see, for example, Steel *et al*, Labib *et al*, Richardson *et al*, and Olsen *et al* 7–10). However, there are also indirect measures of health, such as those that measure the gap between an individual or population’s current health status and ideal health status (living to old age, free from disease and disability), or ‘health lost’ through premature death. Such measures give a different perspective on population health and, in particular, can alter the ways in which we assess and prioritise the importance of pathogenic risk factors or salutogenic characteristics.

Total burden of disease is one such indirect measure of health. In 2018, Scotland published its first estimate of the Scottish Burden of Disease (SBoD), based on a 3 year average of mortality and morbidity from 2013 to 2016.^11^ SBoD is measured in disability adjusted life years (DALYs)—the sum of years of life lost (YLL) and years lived with disability (YLD)—to indicate the burden of disease for an area.^12^ DALYs have been linked to various pathogenic environmental exposures such as air pollution and noise.^13^ The YLL component of the SBoD estimate is the disparity between the actual age at death, and the age to which that person would have been expected to live had death not occurred. The expected lifespan can be determined from life tables. YLL provides a population-level mortality indicator that gives more weight to diseases that cause death at an earlier age.^14^ The SBoD study also identified substantial socioeconomic inequality in health, measuring the socioeconomic situation by small area deprivation.^15^

Measures such as YLL are particularly effective at detecting the impacts of premature poor health and death. Accidents, violence, suicide and substance misuse partially explain the toll on population health at younger ages. Given evidence that natural spaces might be associated with reduction (in risk) of potentially life threatening violence^16^ and suicide,^17^ it would not be far-fetched to consider that such spaces might have some association with an all-cause YLL measure, even with some aspects of natural space being associated with increased premature mortality.^18^

The literature suggests that greenspace may hold beneficial associations for those living in more deprived areas or in lower socioeconomic positions.^19–22^ However, recent evidence shows this may not always be the case and the mediating effect of urban greenspaces to decrease health inequality among different socioeconomic groups may be more important,^23^ which has led to greenspace being identified as ‘equigenic’, promoting health equality. The same causes of death which dominate younger age groups also make substantial contributions to socioeconomic health inequalities. Socioeconomic factors explained 81% (female) and 86% (male) of the local-area variation in premature mortality in the UK.^18^ Given the strong socioeconomic patterning of premature mortality, we hypothesise that natural space might have an equigenic association with YLL.

The first aim of this study was, therefore, to determine whether natural space is associated with YLL in the under 65-year-olds, while accounting for spatial dependency and the deprivation level of the small area unit of measurement. The second aim was to evaluate any moderating effects of natural space on the association between small area deprivation and YLL.

## Methods

### Data sources

#### SBoD data

The outcome data were the YLL component from SBoD 2016, provided at the datazone level separately for males and females under 65 years of age who were resident in Scotland, UK, at time of death. Details on the calculation of YLL (and creation of SBoD) are provided elsewhere,^11 24^ but briefly, the YLL used here is the 3 year average of the discrepancy between expected and actual lifespan of residents who have suffered premature mortality within a datazone.

Datazones are the Scottish government’s preferred small-area statistical reporting units. A datazone is defined as a geographical unit containing approximately 500–1000 household residents, which aligns with physical boundaries and natural communities, has a regular shape and contains households with broadly similar social characteristics.^25^ Other datazone level variables included in the analysis were: the 2016 mid-year estimates of the population of males and females under 65^26^ (used as an offset and for calculating expected counts for modelling); and area deprivation proxied by the income rank component of the Scottish Index of Multiple Deprivation (SIMD) 2016.^27^ Income deprivation captures the proportion of the population in receipt of benefits reflecting low household income and this measure reflects the relative wealth (or lack thereof) of an area’s population. Income rank was divided into quintiles (Q1: lowest income quintile, Q5: highest income quintile) to facilitate easy comparisons between the most and least deprived groups, and to allow for clearer presentation of any potential associations and/or interactions, as well as allowing for non-linear effects. We chose not to include the full SIMD as this included a measure of geographic accessibility and rurality.

#### Natural space data

The main independent variables were percentage cover of natural space within each datazone, with and without including private gardens. Digitised land cover data were obtained from the 2011 Ordnance Survey (OS) MasterMap Topography Layer.^28^ OS MasterMap is the most detailed, accurate, and comprehensive geographical data of the UK’s landscape covering urban and rural areas. As our study covered the whole of Scotland, it was important that natural space coverage in both urban and rural areas was included.

OS grid tiles (20 km) were extracted for the whole extent of Scotland and intersected with datazone geographies using the *sf* package in R.^29^ OS MasterMap contains attributes that were then used to classify landcovers within each datazone that were (1) ‘natural space’, and (2) ‘natural space and private gardens’.

Natural space (NS) features were extracted based on the value of the ‘make’ attribute, which classifies features as man-made, multiple, or natural. Landcovers considered to be natural are those that are not man-made (eg, cliffs, open water, forest) but include landcovers that are anthropogenically modified or managed (eg, cultivated vegetation).

Natural space and private gardens (NSG) features were subset as above (NS) but additionally included features classed with ‘Feature code’ 10 053 (private gardens).

Specifically, the features defined as natural space by OS include: coniferous, deciduous and mixed woodland; scattered trees; scrub; marsh; health; open water (inland or tidal); semi-natural grassland; general natural areas (such as grass on sports pitches, roadside verges and farmland); agriculture; hard bare ground (eg, rocks, boulders, cliffs); and soft bare ground (eg, sand, soil, foreshore).

To create independent variables, the area of each datazone classified as NS and NSG was quantified in square metres and used to calculate the proportional cover of each category by datazone area.

### Statistical analysis

#### Treatment of YLL and distribution by deprivation

YLL is a positive continuous measure, which can also include zeroes. It has a Poisson-like distribution. Following other recent approaches^30^ YLL was rounded before analysis. YLL were considered as the number of observed ‘cases’, and to facilitate direct comparisons at datazone level, observed standardised incidence rates (SIRs) of YLL were calculated (by dividing the observed counts of YLL by the expected counts). These SIRs were first described by deprivation level and percentage of natural space cover.

#### Modelling associations with percentage natural space cover

We examined the structure and distribution of our data and identified that they were over-dispersed. We therefore fitted spatially dependent Poisson generalised mixed models using a Bayesian approach (Integrated Nested Laplace Approximation, INLA). The method chosen was an intrinsic conditional autoregressive model, using a Besag-York-Moillie (BYM) model.^31^ The datazone was treated as an observation level random effect to account for overdispersion, to allow for any variation between datazones,^32^ and to take into account that datazones close to each other might be more similar than those that are further away. Percentage cover of natural space, income deprivation quintile and the interaction between the two were set as main effects.

YLL ‘cases’ were set as the outcome, and the expected number of YLL ‘cases’ in each datazone included as an offset, effectively modelling the SIR of YLL. The output from this model indicated if the incidence (or risk) of YLL for each income quintile and/or level of natural space was greater (an estimate larger than 1.0) than expected, or vice versa, and facilitated direct comparison between datazones with different population sizes. Results are presented as posterior estimates with 95% credible intervals (CrI), which were exponentiated to produce incident rate ratios (IRRs). To aid interpretation of the interaction results, predicted SIRs by income quintile and percentage cover of natural space are presented graphically.

We explored adjusting for urban/rural locality as classified using the Scottish Government’s six-fold urban/rural classification: an analysis of variance (ANOVA) test indicated strong association with percentage natural space, with rural areas having higher mean percentage cover of natural space, as expected. In two datazones, 1e-8 was added where population (and relevant expected values for the INLA models) equalled zero. Zero populations could arise where buildings had been demolished, for example, and where this occurred, the burden of disease for that area was also zero.

All analyses were performed in R version 4.0.5,^33^ using the packages *R-INLA*, *SpatialEpi*, *maptools*, *spdep*, and as a Bayesian analysis with a log link, ‘significance’ is determined by CrI of the posterior estimates that exclude one.

## Results

### Distributions of observed SIRs

**Figure 1:**
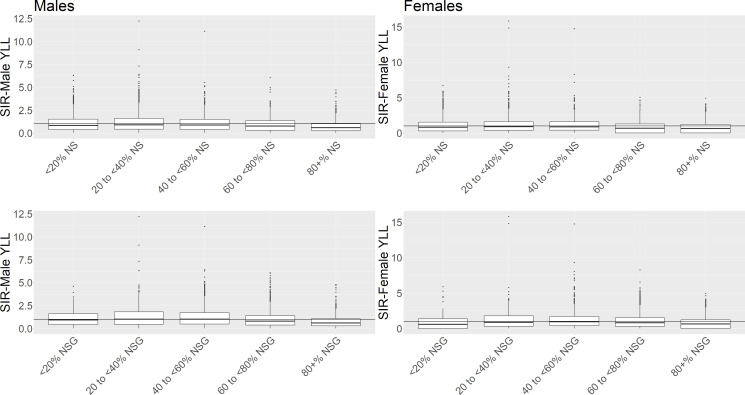
Boxplots of observed standardised incidence rates (SIRs) if years of life lost (YLL) grouped by percentage natural space (%NS) and percentage natural space and gardens (%NSG) for males and females aged under 65. Reference line at SIR=1 denotes standard population SIR. %NS/%NSG, percentage of natural space/natural space and gardens in a datazone.

YLL decreased as deprivation decreased, and decreased overall as the percentage cover of NS increased for both males and females (table 1, figure 1). For NSG, there appeared to be a non-linear trend for both males and females in which the observed SIRs increased with increasing percentage of cover, peaking at 40–60% before decreasing. Examination of figure 1 shows that the observed SIRs are especially variable across the percentages of natural space cover, with outlying rates in every group. The particularly high SIRs were checked for accuracy, and found to be reflective of the data, occurring in datazones with high to medium deprivation (Q1–Q3).

**Table 1:** Medians and interquartile ranges of observed standardised incident rates (SIRs) by income deprivation quintile and percentage cover of natural space (%NS) and natural space and gardens (%NSG). Q1–most deprived

	Male			Female		
	Median	25th percentile	75th percentile	Median	25th percentile	75th percentile
Overall	0.82	0.37	1.45	0.82	0.30	1.52
SIR by income quintile						
Q1	1.53	0.92	2.37	1.42	0.79	2.26
Q2	1.10	0.60	1.69	1.04	0.53	1.78
Q3	0.80	0.39	1.30	0.81	0.34	1.45
Q4	0.56	0.27	0.98	0.61	0.00	1.13
Q5	0.41	0.00	0.81	0.42	0.00	0.91
SIR by NS						
<20% NS	0.84	0.39	1.51	0.82	0.28	1.53
20% to <40% NS	0.90	0.41	1.59	0.90	0.39	1.63
40% to <60% NS	0.88	0.39	1.49	0.88	0.40	1.58
60% to <80% NS	0.78	0.30	1.34	0.68	0.00	1.28
80+% NS	0.60	0.27	1.04	0.65	0.00	1.22
SIR by NSG						
<20% NSG	0.92	0.44	1.60	0.59	0.00	1.33
20% to <40% NSG	1.01	0.45	1.80	0.86	0.31	1.77
40% to <60% NSG	1.02	0.48	1.72	0.95	0.43	1.67
60% to <80% NSG	0.79	0.35	1.39	0.82	0.30	1.51
80+% NSG	0.60	0.27	1.05	0.64	0.00	1.21

NS, natural space; NSG, natural space and gardens; SIR, standardised incident rate.

**Figure 2:**
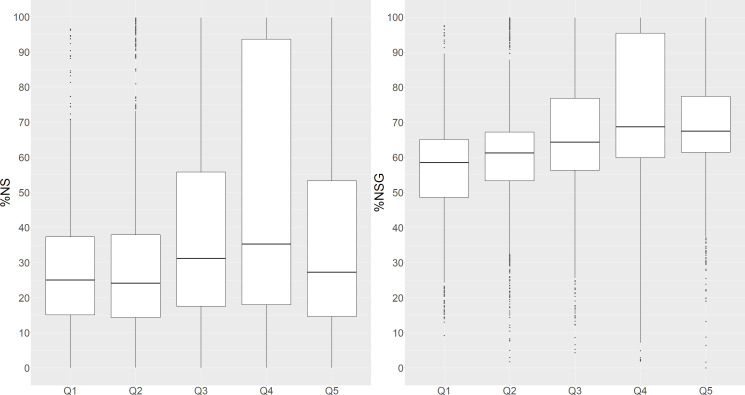
Boxplots of percentage covers of natural space (NS) and natural space and gardens (NSG) by income deprivation quintile. Q1–lowest income quintile.

Figure 2 shows the distribution of natural space by income deprivation. Areas with the highest income deprivation (Q1) have the lowest median percentage cover of natural space and gardens (58.5%, 48.6–65.1%), but Q2 has the lowest cover of NS without gardens (24.1%, 14.3–37.9%). The highest median percentage cover by NS and NSG are in Q3 and Q4. The figure also highlights that high percentages of NS and NSG are considered ‘unusual’ (outliers) in the highest deprivation quintiles Q1 and Q2.

### Modelling associations between percentage natural space cover and YLL (as SIRs)

The output from the spatial BYM model (table 2) supported some of the trends seen with the observed SIRs. Both models (NS and NSG) show an association between income and YLL, with the IRRs decreasing as income deprivation decreased. An association with the percentage of NSG was also present, and of similar magnitude in males (0.993, CrI 0.989 to 0.997) and females (0.993, CrI 0.987 to 0.998); the values indicate that each percent increase in NSG is associated with a 0.7% reduction in IRR. For NS, no association with the outcome of the SIR of YLL was apparent, and the interaction effect absent (CrI included one).

**Table 2:** Summary of BYM models for the association between income quintile and (A) natural space (NS) and (B) natural space and gardens (NSG)

		Males			Females		
		IRR	Lower 95% CrI	Upper 95% CrI	IRR	Lower 95% CrI	Upper 95% CrI
**Model A**							
	(Intercept)	1.373	1.235	1.526	1.180	1.017	1.369
Income quintile	Q1						
	Q2	0.722	0.628	0.831	0.640	0.525	0.779
	Q3	0.484	0.420	0.558	0.459	0.376	0.560
	Q4	0.317	0.275	0.366	0.253	0.206	0.309
	Q5	0.204	0.177	0.235	0.164	0.134	0.201
Percentage NS	%NS	1.000	0.997	1.004	0.997	0.993	1.002
Income interaction with %NS	Q1: %NS						
	Q2: %NS	0.997	0.993	1.001	1.001	0.996	1.007
	Q3: %NS	0.998	0.994	1.002	1.001	0.996	1.006
	Q4: %NS	0.999	0.996	1.003	1.005	1.000	1.010
	Q5: %NS	1.001	0.997	1.005	1.005	0.999	1.010
**Model B**							
	(Intercept)	2.106	1.675	2.648	1.638	1.186	2.261
Income quintile	Q1						
	Q2	0.608	0.442	0.835	0.428	0.273	0.670
	Q3	0.381	0.281	0.516	0.288	0.187	0.442
	Q4	0.217	0.160	0.295	0.125	0.081	0.193
	Q5	0.107	0.076	0.151	0.085	0.052	0.138
Percentage NSG	%NSG	**0.993**	**0.989**	**0.997**	**0.993**	**0.987**	**0.998**
Income interaction with %NSG	Q1: %NSG						
	Q2: %NSG	1.002	0.996	1.007	1.008	1.000	1.015*
	Q3: %NSG	1.003	0.999	1.008	1.008	1.001	1.015
	Q4: %NSG	1.006	1.002	1.011	1.014	1.007	1.021
	Q5: %NSG	1.011	1.006	1.017	1.013	1.005	1.021

*CrI excludes 1.

BYM, Besag-York-Moillie; CrI, credible interval; IRR, incident rate ratio; NS, natural space; NSG, natural space and gardens.

**Figure 3:**
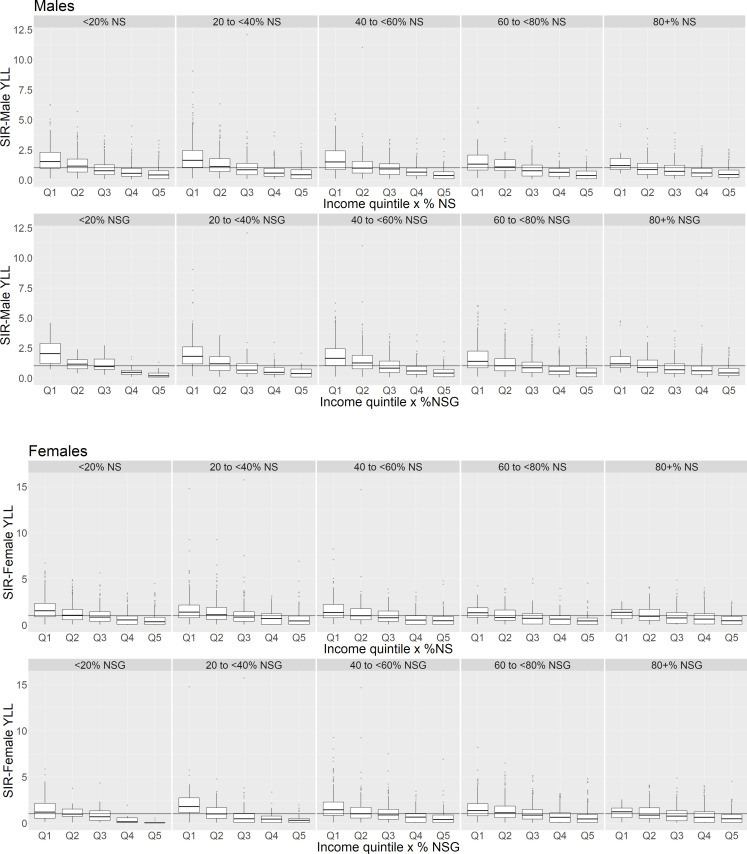
Standardised incident rate (SIR) of years of life lost (YLL) by income quintile for varying levels of natural space (NS), and natural space and gardens (NSG): medians and interquartile ranges. Reference line at SIR=1 denotes standard population SIR. %NS/%NSG, percentage of natural space/natural space and gardens in a datazone. Q1–lowest income quintile (ie, highest income deprivation).

Figure 3 presents graphs of the interaction effects on the predicted SIRs. The outliers present were again examined, and were found to reflect the observed data. The interaction effects between income and natural space also indicate that natural space may reduce inequality between the levels of deprivation (figure 3).

For both males and females, regardless of the quantity of natural space cover present, the predicted SIR in income deprivation quintile Q5 (least deprived) tended to be the lowest, and highest in the most deprived quintile (Q1).

Median SIRs for those in more deprived income quintiles (particularly Q1 and Q2) were lower in areas with more natural space cover compared with areas with the least cover. The predicted median SIR for males in income deprivation Q1 was reduced by around 40% at 80% or more coverage levels: this reduction brought the SIR down to be no different to the standard population level and reduced the inequality between the most and least deprived quintiles.

Even more moderate levels of natural space appear to be able to reduce the inequality in SIRs. In females, the predicted SIR in the most deprived quintile was reduced to be no different from the standard population rate at the presence of at least 40% NSG (as suggested by the overlap of the lower hinge of the boxplots and the reference line in figure 3), thus reducing the disparity in SIR with the lowest deprivation areas. Similarly, the SIR for males in the most income deprived areas is also reduced to a level not different to that of the general population, from at least 40% coverage of NSG, again highlighting a reduction in inequality between the most and least deprived areas.

The model results suggest that the interaction between income and percentage cover of NS is weak/absent; examination of figure 3 also shows a similar trend in the moderating effect of NS as seen for NSG.

## Discussion

### Main findings

Our study aimed to test for an association between natural space and YLL after accounting for deprivation, and for the equigenic association in which inequalities in health appear narrower where natural space exposure is higher. Given the aetiological pathways between green or natural space and better health and the non-health contribution to YLL at younger ages, we hypothesised that associations both between natural space and YLL overall, and inequalities in YLL might be apparent. Our results supported this, showing that YLL decreased overall as the percentage cover of natural space and gardens increased, for both males and females. We also found that an increased amount of natural space within local areas was associated with lower disparity in YLL between the most and least income-deprived areas. The results also indicated that the availability of private garden space was important to health benefits, given that the model with only natural space showed a weaker/absent association with YLL.

### Comparison with existing literature

The results of our study indicate that individuals who were more income deprived had a greater burden of disease as measured using YLL, supporting those presented in the Scottish Burden of Disease 2016: Deprivation Report,^15^ which discusses disparities in overall burden by general (non-income specific) area deprivation. An English analysis of the Global Burden of Disease study from 1990 to 2013 found that YLL decreased by 41% during this time, highlighting a notable improvement in health in England. However, there were stark inequalities in these outcomes between those living in the least and most deprived areas.^34^ An updated analysis of the period 1990 to 2016 found that inequalities between the most and least deprived areas remained and all cause rates of YLL varied by as much as 2× between local areas, depending on socioeconomic status.^7^

We found that increased natural space, including both green and blue spaces, was associated with both reduced YLL and narrow inequalities in YLL between the most and least income deprived areas in Scotland. A recent single city study of Manchester, UK found that a 1-unit increase in neighbourhood greenspace exposure was associated with approximately a 10 year reduction in YPLL.^8^ The leading causes of YLL in England are ischaemic heart disease, lung cancers, cerebrovascular disease and chronic obstructive pulmonary disease.^7^ Previous studies have found people living near greenspace tend to have better mental and physical health.^2^ For example, recent meta-analysis^35^ reports an adjusted odds ratio for cardiovascular mortality of 0.84 (95% CI 0.76 to 0.93) for high versus low greenspace. Modelling of greenspace exposure predicted that the incidence of cardiovascular disease in the least green areas of Norfolk, UK would reduce by 4.8% if they were as green as the average areas in the county.^36^ Further, a study of 31 European counties found that 42 968 deaths could have been prevented during 2015 if the WHO recommended access to green space recommendations were met.^37^ Some pathways that explore how greenspace may improve health include harm-mitigation, restoration and recreation.^38^

The salutogenic potential of natural space may be disproportionately higher in disadvantaged areas.^19^ A recent review describing the potential of greenspaces to reduce health inequalities found greater benefits for those living in the most deprived areas compared with the least deprived for a range of health outcomes, including cardiovascular health and cancers.^22^ Rigolon^22^ *et al* also highlighted that the benefit of greenspace exposure was greater in European studies compared with North American.

In practice, not everyone can live in an area with a high percentage of green or natural space; however, this does not mean that even small amounts of such areas are not beneficial. We found evidence that an increase of 10% natural and garden cover could reduce the incidence rate of YLL by around 7%. Going forward, planning should aim to achieve a good balance of housing and greenspace. For example, the Scottish Government Housing to 2040 policy stipulates that all new housing should provide private gardens,^39^ and other policies promote the retro-fitting of existing provision and re-purposing of vacant and derelict land as community greenspace.

### Strengths and limitations

Our study provides a national level analysis of the YLL data calculated using robust mortality data and life course tables, linked to a national natural space exposure dataset. Both of these datasets are provided by verified government organisations ensuring excellent quality data. By presenting this as an SIR, direct comparisons between datazones can be made, and excess mortality easily identified by values >1; the excess premature mortality faced by those in the most deprived quintiles is clearly evident.

As this is a cross-sectional approach, causality cannot be established. The data are based on all causes of mortality, including factors such as accidents, homicides, and other non-disease specific deaths. Further work could be restricted to premature mortality due to factors that are known to be improved by access to natural space and this might produce even stronger findings. The data are also aggregated to all those under 65 years of age. This may be occluding age-specific trends, and no person specific information, such as lifestyle behaviour and personal economic situation, is known. Finally, rounding of the YLL does lose information. While this study emulated another in using hierarchical Poisson regression, other modelling techniques, such as hurdle models, could be employed.

We were able to measure the proportion of natural space and private gardens within datazones. We did not separate individual typologies, such as tree cover, public parks or blue spaces, which may have provided specific policy recommendations depending on their effect sizes. We were unable to assess whether individuals used their local natural space or the quality of that space.

Our analysis was over a single time period, and we recommend future studies explore natural space exposure and YLL over time.

## Conclusion

Our results suggest that increased natural space is associated with both a reduction in YLL and reduced inequalities in YLL. The apparent health benefits of natural space seem to apply even when indicators sensitive to health events at younger ages are used. An increased amount of natural/green spaces within local areas has the potential to reduce the disparity in YLL between the most and least income deprived areas—the ‘equigenic’ effect. Given the persistent and growing nature of this disparity, such provision and promotion could prove a useful policy lever. Further research should explore this longitudinally and with disease-specific outcomes.

## Data Availability

Data are available upon reasonable request. The data used are available from Ordinance Survey and Scottish Public Health Observatory/Public Health Scotland upon request; data from the latter two are subject to protocol to prevent disclosure of person details.
